# Paracrine Fibroblast Growth Factor 1 Functions as Potent Therapeutic Agent for Intrahepatic Cholestasis by Downregulating Synthesis of Bile Acid

**DOI:** 10.3389/fphar.2019.01515

**Published:** 2019-12-20

**Authors:** Huan Lin, Chuanren Zhou, Yushu Hou, Qi Li, Guanting Qiao, Yang Wang, Zhifeng Huang, Jianlou Niu

**Affiliations:** ^1^ School of Pharmacy, Wenzhou Medical University, Wenzhou, China; ^2^ Engineering Laboratory of Zhejiang Province for Pharmaceutical Development of Growth Factors, Biomedical Collaborative Innovation Center of Wenzhou, Wenzhou, China; ^3^ School of Basic Medical Sciences, Wenzhou Medical University, Wenzhou, China

**Keywords:** fibroblast growth factor 1, bile acid, cholestatic liver disease, alpha naphthylisothiocyanate, negative regulator, intrahepatic cholestasis

## Abstract

Endocrine fibroblast growth factor (FGF) 19 has been shown to be capable of maintaining bile acid (BA) homeostasis and thus hold promise to be a potential therapeutic agent for cholestasis liver disease. However, whether paracrine FGFs possess this BA regulatory activity remains to be determined. In our study, we identified that paracrine fibroblast growth factor 1 (FGF1) was selectively downregulated in the liver of alpha naphthylisothiocyanate (ANIT)-induced intrahepatic cholestasis mice, suggesting a pathological relevance of this paracrine FGF with abnormal BA metabolism. Therefore, we evaluated the effects of engineered FGF1 mutant - FGF1^ΔHBS^ on the metabolism of hepatic BA and found that this protein showed a more potent inhibitory effect of BA biosynthesis than FGF19 without any hepatic mitogenic activity. Moreover, the chronic administration of FGF1^ΔHBS^ protected liver against ANIT-induced injury by reducing hepatic BA accumulation. Taken together, these data suggest that FGF1^ΔHBS^ may function as a potent therapeutic agent for intrahepatic cholestasis liver disease.

## Introduction

As synthesized from cholesterol in the liver, bile acids (BAs) are normally stored in the gallbladder. After meal, it is released into the small intestine to facilitate the digestion and absorption of dietary lipids (de Aguiar Vallim et al., 2013). However, the disturbance of this physiological homeostasis will threaten human health, due to the inherent detergent properties of BAs that are potentially cytotoxic to tissues (Kaplan and Gershwin, 2005).

Cholestasis is one of the most life-threatening liver diseases that is characterized with impaired bile flow and intrahepatic retention of toxic BAs. The occurrence and development of this disease frequently leads to liver fibrosis, cirrhosis and even liver failure (Luo et al., 2014). To date, the main prescribed drugs to treat cholestasis are ursodeoxycholic acid (UDCA) and obelicholic acid (OCA). However, up to 40% of patients’ response poorly to UDCA therapy (Lindor, 2007; Hirschfield et al., 2018). For patients with late-stage cholestasis, the only available option is liver transplantation (Lindor, 2007). Thus, there is still an unmet need for more effective therapeutic agents to relief cholestatic liver disease.

There are accumulating evidences demonstrating that endocrine fibroblast growth factor 15 (FGF15)/FGF19 signals from the intestine to the liver to suppress the expression of cholesterol 7α-hydroxylase (Cyp7A1) (the first rate-limiting enzyme in the classical BA synthetic pathway) as a negative feedback loop on BA synthesis (Inagaki et al., 2005; Goetz et al., 2007; Degirolamo et al., 2016). The discovery of these gut-liver hormonal axes raises the prospect that modulating BA metabolism by FGF1 (Nicholes et al., 2002) may be a novel approach for treating cholestasis. However, the development of a FGF19-based therapy is challenged with regard to its unwanted mitogenic activity and tumorgenicity (Nicholes et al., 2002; Wu et al., 2010). It has been attempted by several groups to uncouple the BA regulatory capacity and tumorigenic activity of FGF19 by engineering a M70 mutant (a FGF19 variant) that retains BA regulatory activity without causing hepatocellular carcinoma (HCC) (Zhou et al., 2014). This nontumorigenic FGF19 variant is shown to reduce hepatic BA accumulation and protect mice from liver injury caused by either intrahepatic or extrahepatic cholestasis, which suggests that it is an applicable approach for the treatment of cholestatic liver disease (Luo et al., 2014; Zhou et al., 2016).

More recently, serum level of FGF21 (another member of FGF endocrine subfamily besides FGF19) is found to be highly upregulated in patients with biliary atresia, suggesting a compensatory response may be initiated (Li et al., 2016). However, the therapeutic effect of this endocrine hormone on cholestasis is still controversial; both its positive and negative regulations on BA synthesis have been reported (Zhang et al., 2017; Chen et al., 2018).

The mammalian FGFs are grouped into five paracrine subfamilies and one endocrine subfamily (Beenken and Mohammadi, 2009; Degirolamo et al., 2016). These FGFs trigger a few common sets of downstream signaling pathways, including PLCγ/PKC, FRS2α/RAS-MAPK, Gab1/PI3 kinase/Akt, and CrkL/Cdc42-Rac (Kouhara et al., 1997; Larsson et al., 1999; Dailey et al., 2005; Eswarakumar et al., 2005; Seo et al., 2009; Chau et al., 2010), *via* activations of FGF receptors (FGFRs) in either an HS-dependent (in the case of paracrine FGFs) or a Klotho coreceptor dependent (in the case of endocrine FGFs) manner (Yayon et al., 1991; Kurosu et al., 2007; Goetz and Mohammadi, 2013). Due to the fact that both endocrine FGF19 and FGF21 are capable of controlling BA metabolism (Inagaki et al., 2005; Zhang et al., 2017; Chen et al., 2018), it is logical to postulate that paracrine FGFs may also possess this ability. Thus, we systematically screened expressions of all paracrine FGFs in alpha naphthylisothiocyanate (ANIT)-induced cholestasis mice and found that only FGF1 was downregulated compared to others, indicating that this paracrine FGF may be associated with the pathogenesis of BA disorders. We further confirmed that FGF1 could suppress BA synthesis both *in vitro* and *in vivo*. Moreover, the chronic administration of FGF1^ΔHBS^ protected liver against ANIT-induced injury by reducing hepatic BA accumulation. Taken together, all the data suggest that FGF1-based therapy may be an effective way to treat cholestatic liver disease.

## Materials and Methods

### Materials

Full-length wide type recombinant human FGF19 (FGF19^WT^) and the FGF1 variant (FGF1^ΔHBS^) carrying triple mutations (Lys127Asp, Lys128Gln, and Lys133Val) were expressed in *E.coli* (BL21) and purified as described in the previous studies (Goetz et al., 2007; Huang et al., 2017). Mouse Total BA Assay Kit was purchased from Crystal Chem INC. Alpha-naphthylisothiocyanate (ANIT) was purchased from Sigma Aldrich (St. Louis, MO, USA). Alanine transaminase (ALT) and aspartate transaminase (AST) Assay Kits were purchased from Sigma Aldrich (St. Louis, MO, USA) and performed in accordance with the protocols provided with manufacturers. All other reagents were of analytical grade.

### Identification of Differential Paracrine FGFs Between ANIT-Induced Cholestasis Model and Vehicle Group

Male C57BL/6J mice (20–25 g) were acquired from the Chinese Academy of Science-Shanghai Laboratory Animal Center. All animals were maintained on a 12-h light/dark cycle and a temperature of 25°C ± 2°C. Animal care and all animal experiments conformed to the Guide for the Care and Use of Laboratory Animals provided by U.S. National Institutes of Health and were approved by the Animal Care and Use Committee of Wenzhou Medical University, China.

ANIT-induced intrahepatic cholestasis was established according to previous reports (Dai et al., 2017). Briefly, mice were randomized into two groups based on body weight. One group was orally administered with a single dose of ANIT (75 mg/kg, dissolved in olive oil), termed as ANIT-induced cholestasis model group; another group was treated with the same volume of olive oil, serving as the vehicle group. Mice were euthanized 48 h after administration. Blood of mice was collected to determine serum levels of liver enzymes (ALT and AST). Liver tissues of both groups were collected and total RNA was isolated using Trizol reagent (Invitrogen) according to the manufacturer’s instructions. The differential mRNA levels of all paracrine FGFs between ANIT-induced cholestasis model and the vehicle group were determined by RT-PCR and relative mRNA levels were calculated by comparative threshold cycle method using β-actin as the internal standard. The primers of all paracrine FGFs were listed in the (**Table S1**).

### Primary Hepatocyte Experiments

Primary mouse hepatocytes were isolated and cultured according to the methods described previously (Zhou et al., 2017). Primary mouse hepatocytes were plated on rat tail collagen-coated dishes at a density of 1.5×10^5^ cells/cm^2^ and were allowed to attach for 24 h in a 37°C incubator with 5% CO_2_. Culture medium was consisted of 2% fetal bovine serum Williams’ E medium supplemented with 10 nM dexamethasone, 1% penicillin/streptomycin, L-glutamine. Twenty-four hours after isolation, cells were treated with the indicated concentration of FGF1^ΔHBS^ or FGF19^WT^ (0.2, 2.0, or 20 nM) for 6 h. Total RNA was isolated using Trizol reagent (Invitrogen) according to the manufacturer’s instructions. The relative mRNA levels of Cyp7A1, Cyp8B1, Cyp27A1, and Cyp7B1 were determined by real-time polymerase chain reaction (RT-PCR).

For knockdown of FGF receptor 4 (FGFR4) expression by siRNA, cells were seeded in 6-well plates for 24 h. Transient transfections were performed using Lipofectamine RNAiMAX (Thermo Fisher Scientific, Waltham, MA) according to manufacturer’s protocol. After transfection with either siRNA (control or FGFR4 siRNA) (all from Santa Cruz Biotechnology, Dallas, Texas, USA) for 24 h, cells were treated with FGF1^ΔHBS^ (20 nM) for 6 h. Then, cells were extracted and analyzed the Cyp7A1 mRNA levels by RT-PCR.

### Acute Effects of FGF1^ΔHBS^ on the Genes That Involved in the BA Metabolism

To directly and comprehensively evaluate the effects of FGF1^ΔHBS^ on BA metabolism, 6–8 weeks’ C57BL/6J mice were administered with a single intraperitoneal injection of PBS, FGF1^ΔHBS^, or FGF19^WT^ at increasing doses (0.01, 0.1, and 1.0 mg/kg) respectively. After 4 h of administration, liver tissues were collected and snap-frozen in liquid nitrogen for analysis of the key genes involved in BA metabolism including BA biosynthesis and transport.

### Effects of Subchronic Administration of FGF1^ΔHBS^ on the BA Homeostasis

A 6-day subchronic study was performed in 6–8-week-old C57BL/6J mice. Male mice were administered with vehicle, FGF1^ΔHBS^ (0.1 and 1.0 mg/kg, once daily) or FGF19^WT^ (0.1 and 1.0 mg/kg, twice daily BID), PBS and FGF19^WT^-treated mice served as negative and positive control respectively. On the sixth day, the mice received the last dose and were placed in a new cage without food. Tissue samples including liver, gallbladder, and small intestine were collected 4 h after the last administration for genes and BA content analysis. Collect the small intestine and retain the complete contents.

Total BAs of liver, gallbladder, and small intestine were determined using Mouse Total BA Assay Kit (Crystal Chem INC). The extraction method from tissues was according to methods described previously (Chen et al., 2018). Briefly, the froze liver, gallbladder, and small intestine from subchronic treated mice were individually homogenized with 75% ethanol and total BAs were extracted by incubating the tissue homogenate in a 50°C shaker for 2 h. Supernatants from the extraction were collected after centrifugation and diluted in PBS for analysis. The dilution factors for each tissue extract were estimated to ensure that BA measurements fell in the linear range of the standard curve using mouse BA kit. The BA pool size was determined as sum of the amount of total BAs in the liver, gallbladder, and small intestine and its contents.

### Real-Time Polymerase Chain Reaction (RT-PCR)

Total RNA was isolated from treated primary hepatocytes or frozen tissues, such as the liver, intestine, and gallbladder using TransZol Up Kit (TransGen Biotech). The total RNA was reverse-transcribed into complementary DNA with One-Step gDNA Removal Kit (TransGen Biotech). Quantitative real-time polymerase chain reaction (RT-PCR) was done using ChamQ Universal SYBR qPCR Master Mix (Vazyme) with specific primers (listed in the **Table S1**) on a Step One Plus Real-Time PCR system (Applied Biosystems^®^ Quant Studio^®^ 3). β-actin was used as an endogenous control to normalize for differences in the amount of total RNA added to each reaction. For mRNAs levels of paracrine FGFs with the cycle threshold (CT) value equal to or greater than 35 are considered undetectable.

### Western Blot Analysis of Protein Expression

Liver tissues were then homogenized in protein extraction buffer (Sigma-Aldrich, St. Louis, MO) with protease and phosphatase inhibitors, and collected by centrifuging. The protein concentration was determined using a bicinchoninic acid (BCA) Protein Assay Kit (Thermo Scientific, Waltham, MA), and equal amounts of samples were mixed with loading buffer, subjected to SDS-polyacrylamide gels at 110 V for 100 min, and then the protein was transferred onto polyvinylidine fluoride membranes (PVDF) (Millipore-Billerica, MA, USA). After blocking with 5% nonfat milk for 2 h at room temperature, the PVDF membranes were washed three times with Tris-buffered saline (pH 7.6) containing 0.1% Tween-20 (TBST) and then incubated at 4°C overnight with primary antibody to mouse FGF1 (1:1000) or glyceraldehyde-3-phosphate dehydrogenase (GAPDH) (1:2000), all the antibodies were obtained from Abcam (Cambridge, MA). The membranes were washed and later incubated with HRP-conjugated secondary antibody. Immuno-reactive protein bands were visualized using the ChemiDocTM XRS with Imaging LabTM Software (Bio-Rad, Hercules, CA) and quantified by optical densitometry using Image J software (National Institutes of Health, Bethesda, MD, USA).

### Therapeutic Effects of FGF1^ΔHBS^ on the ANIT-Induced Interhepatic Cholestasis

Male C57BL/6J mice (6–8 weeks old; n = 10 mice per group) were injected intraperitoneally with PBS, FGF1^ΔHBS^ (0.1 mg/kg) or FGF1^ΔHBS^ (1.0 mg/kg) once daily for 6 days. On the fourth day, a single dose of ANIT (75 mg/kg, dissolved in olive oil) was administered *via* oral gavage. Mice were euthanized on the sixth day, 4 h after the final dosage. Serum, liver, gallbladder, and intestine samples were collected for analysis. Perform the analysis of serum liver function index (ALT and AST). Detect the BA content in serum, liver, gallbladder, and small intestine.

### Histological Analysis

Hematoxylin and Eosin (H&E) staining was performed to detect the protection of FGF1^ΔHBS^ for liver injury in the ANIT-induced intrahepatic cholestasis. Briefly, liver tissues of each group were collected and kept in 4% paraformaldehyde for a post-fix overnight, and the paraffin-embedded liver tissues were then sectioned into 5 μm thickness sections by LEICA RM2235 (LEICA, Germany). The sections were dried at 40°C, deparaffinized, and then rehydrated using a series of ethanol solutions. The rehydrated sections were subjected to H&E solution based on the manufacture’s protocol. After the slides were gently washed with tap water and subsequently rinsed in distilled water, the slides were mounted with mounting medium. Finally, the sections were observed and imaged using a Nikon ECLIPSE NI fluorescence microscope (Nikon, Japan).

### Statistical Analysis

All results are expressed as means ± SEM. One-way ANOVA followed by Dunnett’s posttest was used to compare data from multiple groups (GraphPad Prism). When indicated, Student’s t test or Mann-Whitney test was used to compare two treatment groups. A P-value of 0.05 or less was considered statistically significant.

## Results

### FGF1 Is Selectively Downregulated in the Liver of ANIT-Induced Intrahepatic Cholestasis Mice

**Figure 1:**
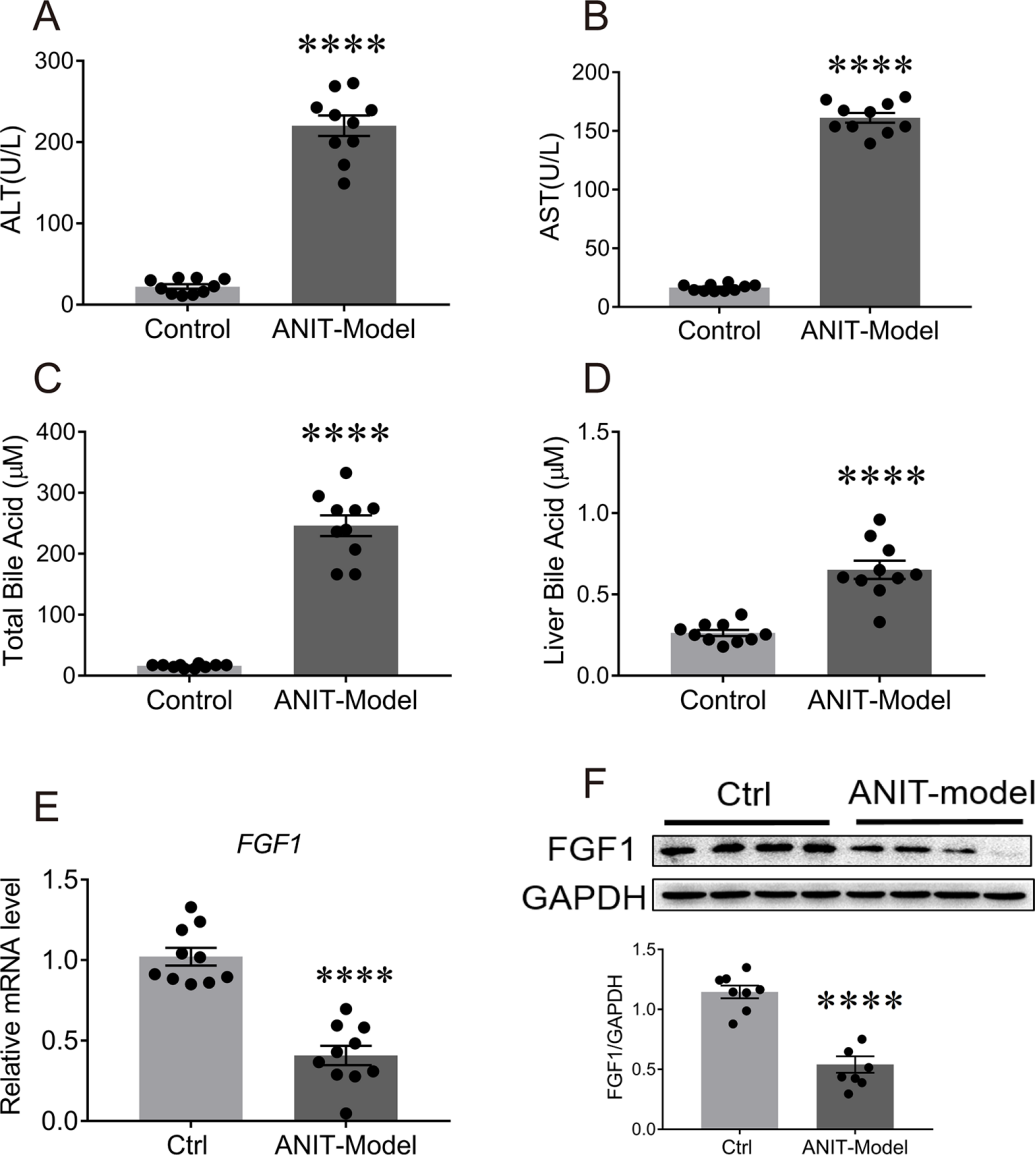
The mRNA and protein levels of FGF1 in liver were downregulated in the ANIT-induced intrahepatic cholestasis mouse model. Eight-week-old C57BL/6J mice were orally administrated with olive oil (control group) or 75 mg/kg ANIT (cholestasis mouse model) for once and then liver tissues were collected and analyzed after 48 h. **(A**, **B)** Evaluation of liver function as determined by serum levels of ALT and AST. **(C**, **D)** Serum BA concentrations **(C)** and hepatic BA pool **(D)** of each group were measured. **(E)** The levels of hepatic FGF1 gene expression of each group were examined by real-time polymerase chain reaction (RT-PCR); **(F)** Representative western blot analysis (up panel) and densitometric quantification (down panel) of FGF1 protein expression in liver tissues from ANIT-induce intrahepatic cholestasis mouse model and control group; data are normalized to GAPDH. Data are presented as mean ± SEM; ****p < 0.0001 versus control group; n = 8-12. ANIT-model, ANIT-induced intrahepatic cholestasis mouse model; Ctrl, control group; FGF1, fibroblast growth factor 1; GAPDH, glyceraldehyde-3-phosphate dehydrogenase.

To explore the pathological relevance of paracrine FGFs with cholestatic liver disease, C57BL/6J mice were orally challenged with a single dose of anaphthylisothiocyanate (ANIT - a xenobiotic compound) (75 mg/kg of body weight) for 48 h to induce intrahepatic cholestasis with olive oil as (Yu et al., 2017). We found that serum levels of ALT, AST, and total BA were markedly upregulated with concomitant increases of hepatic BAs in these mice (**Figures 1A**, **D**). To our surprise, we found that only mRNA level of FGF1 in the liver was selectively downregulated (51% reduction relative to vehicle group) compared to other paracrine FGFs (**Figure 1E**; **Table S2**). This was further confirmed by the fact that the protein expression of hepatic FGF1 was also significantly abrogated (**Figure 1F**). Taken together, these results suggest that FGF1 may be involved in the control of BA metabolism.

### FGF1^ΔHBS^ Suppresses Biosynthesis Pathways of BA in Primary Mouse Hepatocyte

Considering the potential safety of wild type FGF1 (FGF1^WT^), we used an FGF1 variant (FGF1^ΔHBS^) in the following study. As reported in our previous study, this mutant exhibits a markedly reduced proliferative potential while preserving the full metabolic activity of FGF1^WT^ (Huang et al., 2017).

The synthesis of BAs in the liver are tightly controlled by two pathways to match the physiological needs, which are comprised of one classic pathway catalyzed by Cyp7A1 and an alternative pathway regulated by two key enzymes (Cyp27A1 and Cyp7B1) (Russell, 2009).

**Figure 2:**
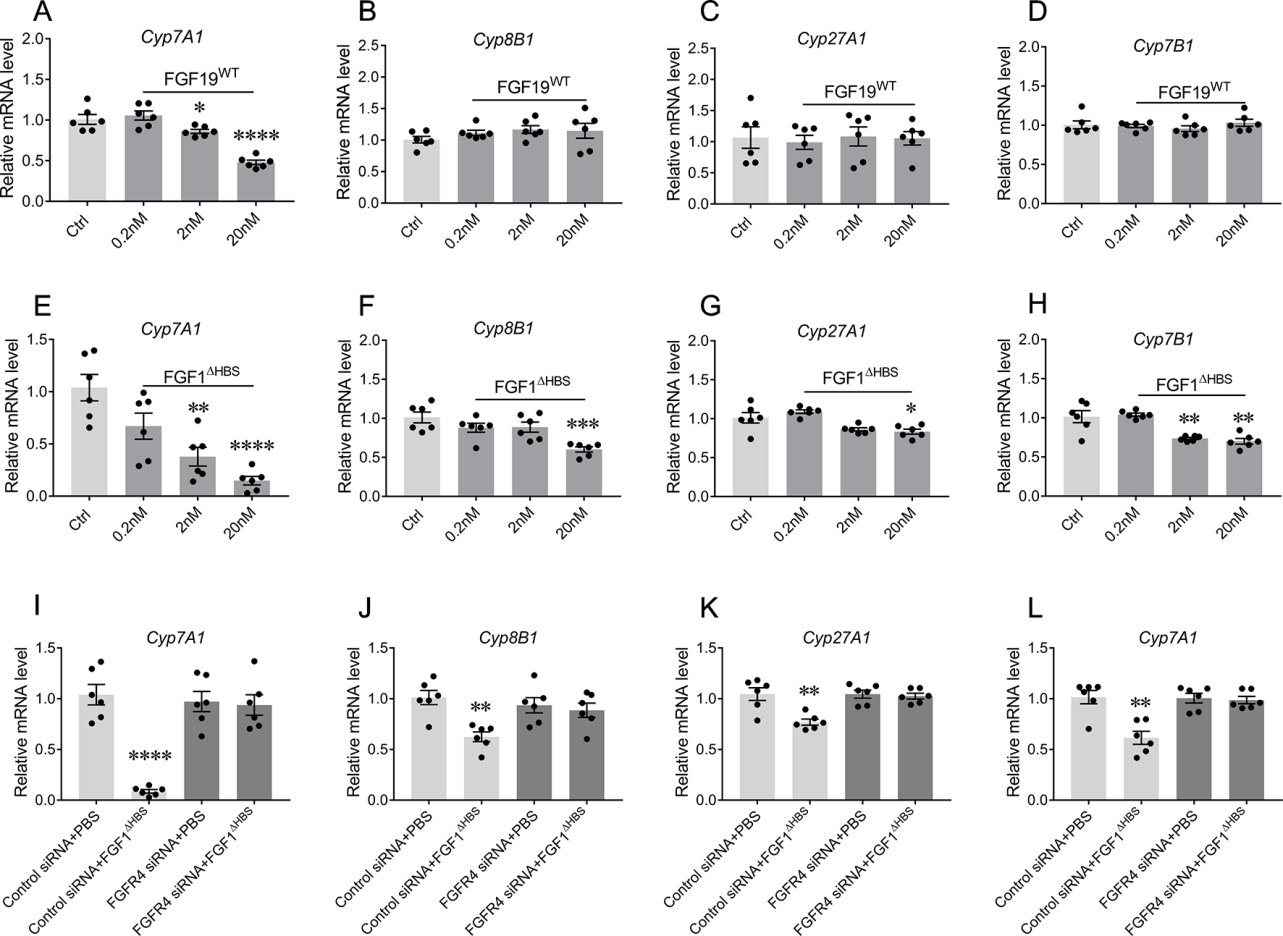
The bile acid (BA) regulatory activity of FGF1 in primary mouse hepatocytes. **(A**-**H)** Primary mouse hepatocytes were stimulated by different concentrations of FGF19^WT^ **(A**-**D)** or FGF1^ΔHBS^ **(E**-**H)**, relative mRNA levels of Cyp7A1, Cyp8B1, Cyp27A1, and Cyp7B1 were determined by real-time polymerase chain reaction (RT-PCR) (n = 6) and normalized to actin mRNA levels. **(I**-**L)** Primary mouse hepatocytes with and without knockdown FGFR4 were stimulated by PBS or 20 nM FGF1^ΔHBS^, relative mRNA levels of Cyp7A1, Cyp8B1, Cyp27A1, and Cyp7B1 were determined by RT-PCR and normalized to actin mRNA levels. Data are expressed as fold change relative to control group (PBS-treated primary mouse hepatocytes). Data are presented as mean ± SEM; ****p < 0.0001 versus control group. *P < 0.05; **P < 0.01; ***P < 0.001.

To assess the capability of FGF1^ΔHBS^ to regulate the biosynthesis of BA, primary mouse hepatocytes were stimulated with increasing concentrations of FGF1^ΔHBS^ with vehicle and FGF19^WT^ as controls. Consistent with previous reports (Holt et al., 2003; Luo et al., 2014), FGF19 selectively inhibited the mRNA levels of Cyp7A1 (52% reduction verse vehicle group) in the primary mouse hepatocytes without affecting those of Cyp8B1, Cyp27A1, and Cyp7B1 (**Figures 2A**–**D**). Notably, FGF1^ΔHBS^ could inhibited the mRNA levels of Cyp7A1, Cyp8B1, Cyp27A1, and Cyp7B1, showing a greater potency on the inhibition of Cyp7A1 mRNA levels compared to that FGF19^WT^ (**Figures 2E**–**H**) and these inhibitory effects were compromised by the knockdown of FGF receptor 4 (FGFR4) expression by siRNA transfection (**Figures 2I**–**L**). Taken together, these data indicate that FGF1^ΔHBS^ directly downregulates BA biosynthesis by FGFR4-mediated inhibiting of the classical and alternative pathways.

### FGF1^ΔHBS^ Acutely Inhibits BA Synthesis Pathways *In Vivo*

**Figure 3:**
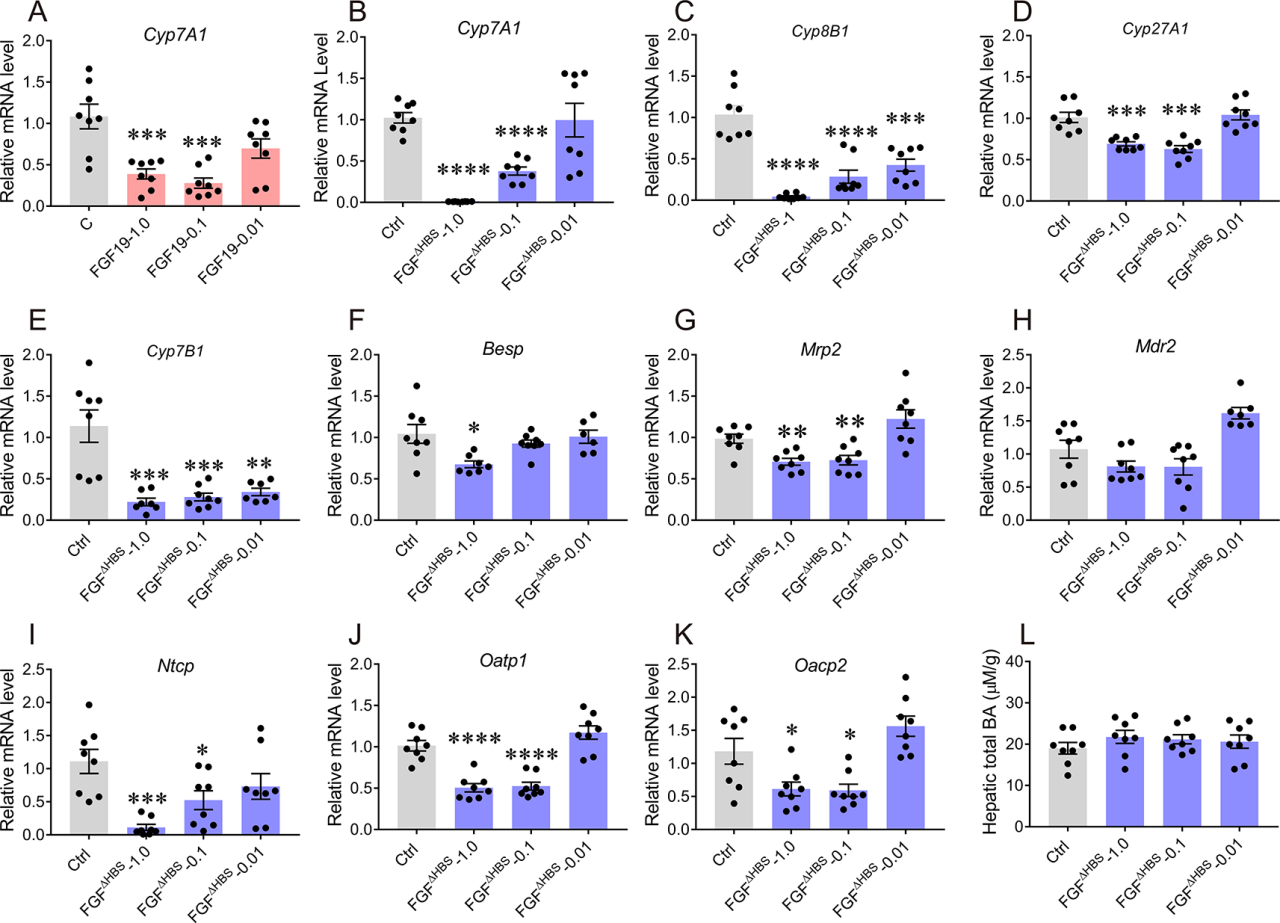
Acute effects of FGF1^ΔHBS^ on the expression of hepatic genes involved in bile acid (BA) homeostasis. Eight-week male C57BL/6J mice were injected intraperitoneally with FGF1^ΔHBS^ or FGF19^WT^ at dose of 0.01, 0.1 and 1.0 mg/kg, FGF19^WT^-treated mice served as a positive control. Liver tissues were collected 4 h after dosing and the hepatic genes involved in BA biosynthesis and transporter were evaluated by real-time polymerase chain reaction (RT-PCR). **(A)** Effect of FGF19^WT^ on Cyp7A1 mRNA; **(B**–**E)** Hepatic mRNA levels of Cyp7A1 **(B)**, Cyp8B1 **(C)**, Cyp27A1 **(D)**, and Cyp7B1 **(E)** were determined by RT-PCR. **(F**–**H)** RT-PCR analysis of canalicular efflux transporters (Bsep, Mrp2 and Mdr2). **(I**–**K)** RT-PCR analysis of basolateral uptake transporters (Ntcp, Oatp1, and Oatp2). **(L)** Hepatic BA pool after acute administration of FGF1^ΔHBS^. Data was normalized to actin mRNA levels and expressed as fold change relative to control group (PBS-treated mice). Data are presented as mean ± SEM; *P < 0.05; **P < 0.01; ***P < 0.001; **** p < 0.0001 versus control group; n = 8.

**Figure 4:**
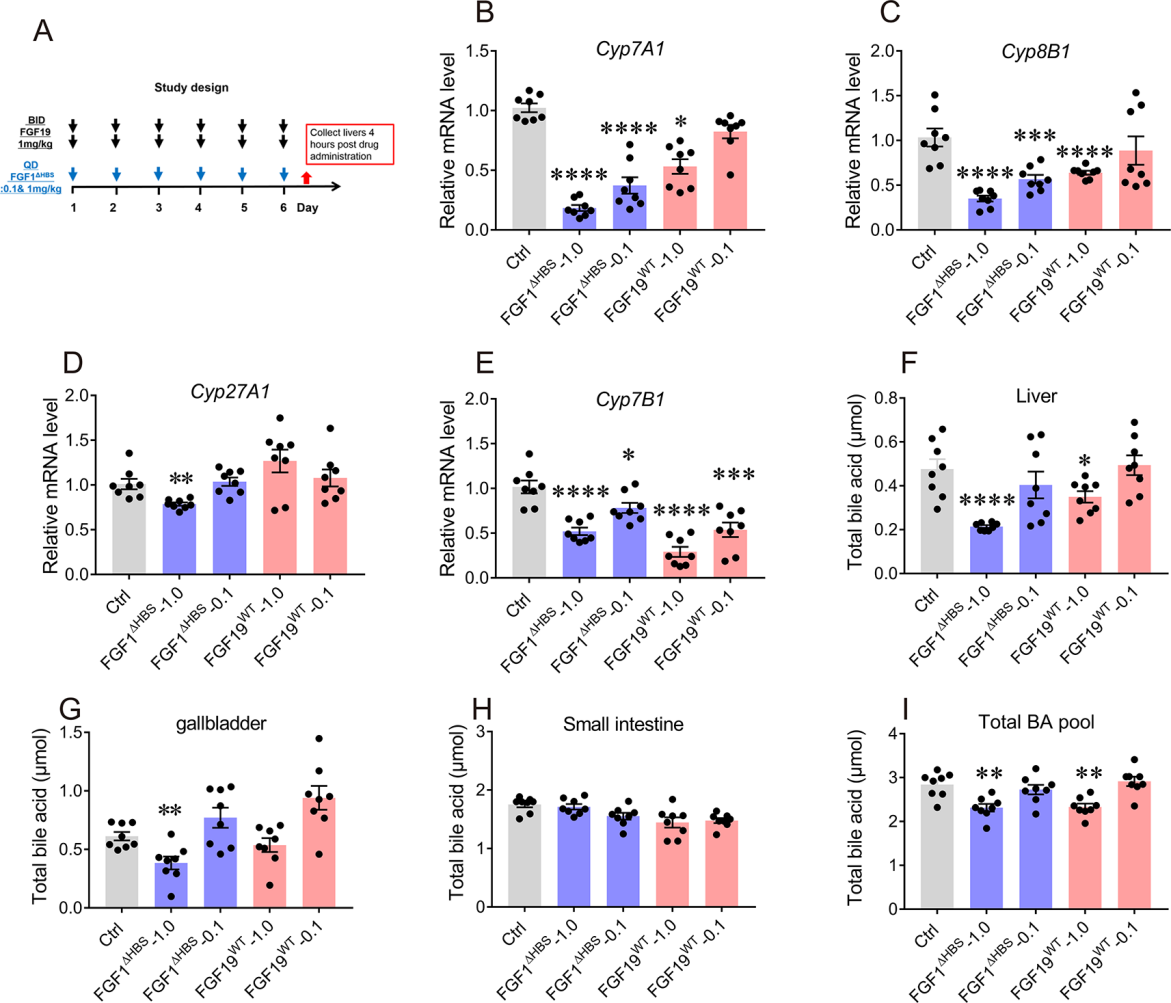
Effects of chronic FGF1^ΔHBS^ and FGF19^WT^ administration on hepatic bile acid (BA) synthetic enzyme and BA pool of liver, small intestine, and gallbladder in normal mice. **(A)** Male C57BL/6J mice were treated at indicated doses for 6 days and tissues were collected for analysis 4 h after administration. **(B**-**E)** The effect of chronic FGF1^ΔHBS^ and FGF19^WT^ treatment on the mRNA levels of Cyp7A1 **(B)**, Cyp8B1 **(C)**, Cyp27A1 **(D),** and Cyp7B1 **(E)** in the liver of C57BL/6J mice. **(F**-**I)** BA pool of liver (F), small intestine (G), and gallbladder (H). Total BA pool size (I) were calculated. Data are presented as mean ± SEM; *P < 0.05; **P < 0.01; ***P < 0.001; ****p < 0.0001 versus Control group; n = 8.

To evaluate the inhibitory effect of FGF1^ΔHBS^ on hepatic BA synthesis *in vivo*, C57BL/6J mice were received with a single intraperitoneal (i.p.) injection of FGF1^ΔHBS^ at the dose of 0.01, 0.1 or 1.0 mg/kg of body weight. As previous reported (Luo et al., 2014), FGF19^WT^ remarkedly suppressed the expression of Cyp7A1 mRNA levels 4 h after injection (1.0 mg/kg, 39% reduction relative to vehicle group) (**Figure 3A**). In the case of FGF1^ΔHBS^, its acute administration dose-dependently inhibited hepatic mRNA expressions of Cyp7A1, Cyp8B1, Cyp27A1, and Cyp7B1 (**Figures 3B**–**E**), suggesting that FGF1^ΔHBS^ might reduce BA biosynthesis through both the classic and alternative pathways, which was consistent with the *in vitro* data. Notably, FGF1^ΔHBS^ more potently inhibited the mRNA level of Cyp7A1 than FGF19^WT^ (99% reduction relative to vehicle group at 1.0 mg/kg dose), probably due to that FGFR4-binding affinity of FGF1^ΔHBS^ is much higher than that of FGF19^WT^ (**Figure 4B**).

In addition to BA synthesis, the maintenance of BA homeostasis relies on various hepatic membrane transporters (Zollner et al., 2006; Halilbasic et al., 2013). In the liver, the uptake of BA from hepatic portal vein is mainly mediated by basolateral uptake transporters (Ntcp, Oatp1, and Oatp2), while its excretion through microbilliary ducts is regulated by BA canalicular efflux transporters (canalicular bile salt export pump Bsep, Mrp2, and Mdr2) (Zollner et al., 2006; Halilbasic et al., 2013). To comprehensively investigate the effects of FGF1^ΔHBS^ on BA intracellular and extracellular transports, mRNA levels of both basolateral uptake transporters and BA canalicular efflux transporters in the liver were determined. As shown in **Figures 3F**–**H**, expressions of Bsep and Mrp2 were only modestly suppressed by FGF1^ΔHBS^ treatment without affecting that of Mdr2. For basolateral uptake transporters, FGF1^ΔHBS^ significantly reduced mRNA levels of Ntcp, Oatp1, Oacp2, and especially Ntcp (89% reduction verse vehicle group), indicating that FGF1^ΔHBS^ may prevent the uptake of BA from hepatic portal vein (**Figures 3I**–**K**). Moreover, the measurement of hepatic BA pool showed that there was no remarkable difference between FGF1^ΔHBS^-treated mice with control group (**Figure 3L**). Taken together, these data suggest that FGF1^ΔHBS^ is a more potent inhibitor on BA biosynthesis than FGF19 *in vivo* and prevent the uptake of BA from hepatic portal vein.

### Chronic Administration of FGF1^ΔHBS^ Inhibits Biosynthesis of BA Without Inducing Hepatic Proliferation

A 6-day study was conducted to evaluate the long-term effect of FGF1^ΔHBS^ on BA biosynthesis. The dosage and frequency used was according to previous report (Chen et al., 2018), mice were i.p. administered with FGF19^WT^ (twice daily) or FGF1^ΔHBS^ (once daily) at 0.1 or 1.0 mg/kg of body weight (**Figure 4A**). Consistent with its acute effects, FGF1^ΔHBS^ at 1.0 mg/kg significantly inhibited hepatic expressions of Cyp7A1, Cyp8B1, Cyp27A1, and Cyp7B1 (**Figures 4B**–**E**) with a remarkable reduction of BA content in the liver and gallbladder (**Figures 4F**–**H**). Besides, total BA pool was also largely reduced (**Figure 4I**). These data further demonstrate that FGF1^ΔHBS^ can inhibit BA biosynthesis and reduce BA pool by downregulating BA synthetic pathways. Thus, we speculate that FGF1^ΔHBS^ may be a potential therapeutic agent for treating cholestasis.

**Figure 5:**
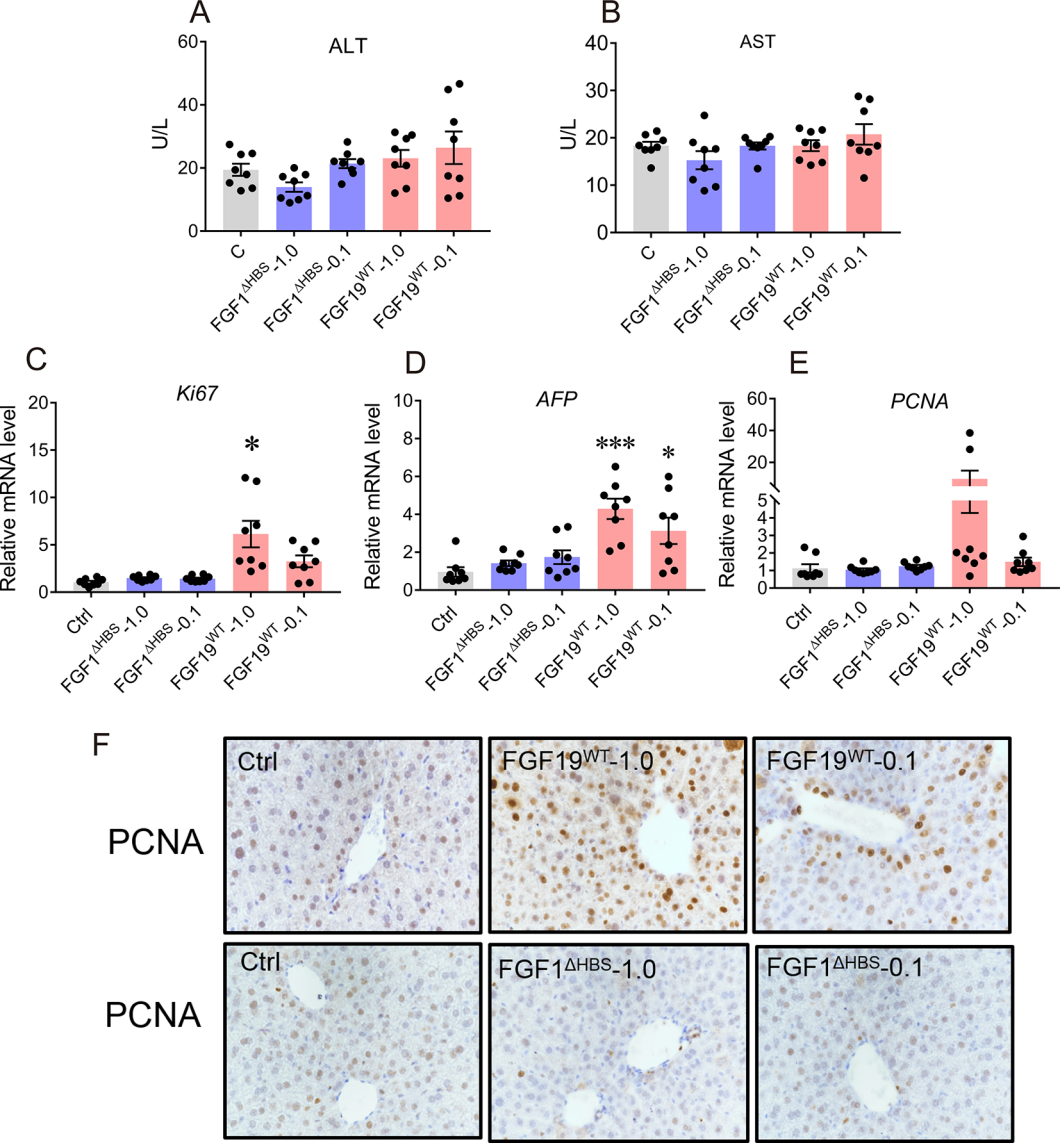
Safety evaluation of chronic FGF1 administration. **(A**, **B)** Evidence of liver injury as determined by serum activities of liver enzymes Alanine transaminase and aspartate transaminase (ALT and AST). **(C**–**E)** Real-time polymerase chain reaction (RT-PCR) analysis of hepatic mRNA levels of the proliferation markers including Ki-67, AFP, and PCNA in C57BL/6J mice after chronic treatment, data was normalized to actin mRNA levels and expressed as fold change relative to control group (PBS-treated mice) **(F)** immune-histochemical staining using PCNA. Data are presented as mean ± SEM; *P < 0.05; ***P < 0.001; versus control group; n = 8.

In addition, we evaluated the safety of chronic FGF1^ΔHBS^ treatment. Serum levels of ALT and AST were not altered by FGF1^ΔHBS^, suggesting that liver was not injured (**Figures 5A**, **B**). We further assessed the mitogenic activity of FGF1^ΔHBS^ and FGF19^WT^ in the 6-day study by determining the hepatic mRNA levels of proliferation markers including Ki67, alpha fetaprotein (AFP), and proliferating cell nuclear antigen (PCNA). Consistent with previous studies (Wu et al., 2010), FGF19^WT^ significantly upregulates these proliferating signals in the liver (**Figures 5C**–**E**). However, mice treated with FGF1^ΔHBS^ did not exhibit any hepatic proliferation activity (**Figures 5C**–**E**). Moreover, signs of hyperplasia by immune-histochemical staining using PCNA showed that FGF19^WT^ caused a clear increase in the number of hyperproliferating cells in the liver of mice. As expected, there was no increase in the number of hyperproliferating cells in the livers of FGF1^ΔHBS^-treated mice over that of the PBS-treated control (**Figure 5F**). This consistence with our previous study further confirms the safety of chronic FGF1^ΔHBS^ treatment.

### FGF1^ΔHBS^ Ameliorates ANIT-Induced Intrahepatic Cholestasis

**Figure 6:**
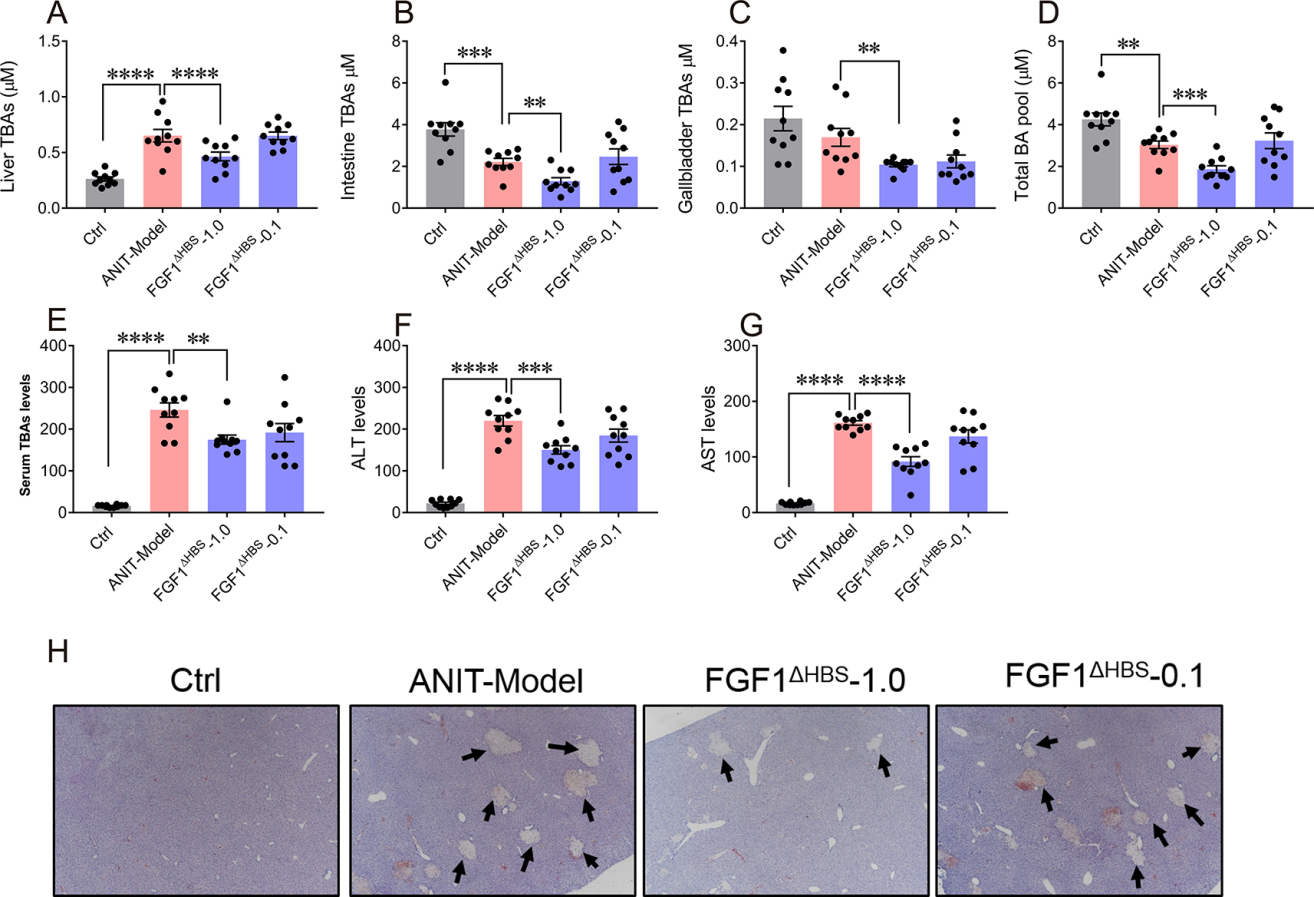
FGF1^ΔHBS^ protects mice from ANIT-induced intrahepatic cholestasis. Male C57BL/6J mice were intraperitoneally injected by PBS or FGF1^ΔHBS^ (n = 10), for 4 days. ANIT in olive oil (75 mg/kg) was administered orally on the fourth day, and protein dosing was continued for another 2 days. **(A**-**D)** Size of BA pools from the liver **(A)**, intestine **(B)**, and gallbladder **(C)**. Total BA pool sizes **(D)** were calculated. **(E**-**G)** Analysis of biochemical parameters indicative of liver damage including serum total BA **(E)**, Alanine transaminase (ALT)_ **(F)**, and aspartate transaminase (AST) **(G)** levels. **(H)** Representative H&E-stained liver sections of each group. **P < 0.01; ***P < 0.001; ****P < 0.0001.

We further assessed the potential therapeutic efficacy of FGF1^ΔHBS^ on ANIT-induced intrahepatic cholestasis. Consistent with previous studies, increased intrahepatic BA content and reduced the intestine BA pool were observed in the mice treated by ANIT, compared with that of the vehicle group, confirming the establishment of intrahepatic cholestasis model (**Figures 6A**–**D**). Notably, we observed that FGF1^ΔHBS^ (1.0 mg/kg of body weight) treatment significantly reduced BA contents in the liver, gallbladder, and small intestine and thus lowered the total BA pool (**Figures 6A**–**D**).

The elevated hepatic BA accumulation in ANIT-induced intrahepatic cholestasis mice induced a remarkable liver damage as revealed by increasing serum levels of ALT and AST (**Figures 6E**–**G**). We found that the chronic treatment of FGF1^ΔHBS^ (1.0 mg/kg of body weight) significantly lowered serum levels of total BA, ALT, and AST, indicating a protective effect of FGF1^ΔHBS^ on ANIT-induced liver damage. The histological analysis suggested that FGF1^ΔHBS^ treatment reduced cholestasis associated necrotic lesions in the liver, compared to the vehicle treatment (**Figure 6H**). Taken together, all the data demonstrate that FGF1^ΔHBS^ protects against liver damage in ANIT-induced intrahepatic cholestasis by reducing hepatic BA accumulation through the inhibition of both the classic and alternative pathways.

## Discussion

The homeostasis of BA metabolism is tightly regulated by numerous enzymes, transporters, and nuclear receptors (Halilbasic et al., 2013). Previous studies have shown that both FGF19 and FGF21 are capable of controlling its metabolism (Schaap et al., 2009; Luo et al., 2014; Wunsch et al., 2015; Zhang et al., 2017; Chen et al., 2018). Here, we firstly identified that paracrine FGF1 was selectively downregulated in the liver of ANIT-induced intrahepatic cholestasis mice (**Figures 1E**–**G**). Then, we confirmed that the engineered FGF1^ΔHBS^ could suppress BA biosynthesis by downregulating both of the classical and alternative pathway *in vitro* and *in vivo* (**Figures 2** and **3**). We further demonstrate that exogenous administration of FGF1 could protect ANIT-induced cholestasis liver injury by inhibiting biosynthesis of BA (**Figures 2**–**4**).

FGF1 is a prototype of FGF family that has been implicated in various physiological processes including wound healing, neuroprotection and adipogenesis (Beenken and Mohammadi, 2009). Recently, Johan W. Jonker et al. identified that PPARγ-FGF1 axis is critical for maintaining metabolic homeostasis and insulin sensitization (Jonker et al., 2012). Although known as a mitogenic factor, exogenous administration of FGF1 results in potent and insulin-dependent glucose lowering effect in insulin resistance mice. This discovery uncovers an unexpected, neomorphic insulin-sensitizing effect of FGF1 to treat type 2 diabetes (Suh et al., 2014) and expands the functions of this classically known mitogen. In our study, we firstly show another novel function of FGF1 to protect cholestasis liver disease by regulating BA biosynthesis.

The signaling and functions of FGFs are tightly regulated by spatial and temporal expressions of FGFs, FGFRs, and coreceptors (heparin sulfate and klotho) and most importantly by binding specificity of FGF-FGFRs (Beenken and Mohammadi, 2009). As a unique member of FGFs, FGF1 is termed as “the universal FGFR ligand” because it overrides the barrier of FGF-FGFRs binding specificity (Beenken et al., 2012), which makes it binding with liver-enriched FGFR4 and forming the structural basis for BA regulatory activity (**Figure 2**). Notably, the engineered FGF1 partial agonist (FGF1^ΔHBS^) carrying triple mutations located on the heparin sulfate binding regions, which diminishes the ability of FGF1^ΔHBS^ to induce heparin sulfate-assisted FGFR dimerization (Huang et al., 2017) but did not affect its binding specificity with FGFRs. This may be the reason that FGF1^ΔHBS^ still exhibits BA regulatory activity (**Figures 2** and **3**).

One plausible explanation for FGF1^ΔHBS^ not inducing hepatic proliferation may rely on the “threshold model” published in our previous study; as the dimerization strength of FGFR decreases, the mitogenic potential of FGF1 is dissipated much earlier before the decrease of its glucose-regulatory activity. Thus, the mitogenic and glucose-lowering activity of FGF1 could be uncoupled by dampening FGF-FGFR dimerization through triple mutations that diminish FGF1-heparin binding affinity (Huang et al., 2017). In the study, we found that FGF1^ΔHBS^ could regulate BA metabolism without exhibiting any hepatic proliferation activity, suggesting that the BA regulatory activity and mitogenic activity of FGF1 could be also separated in this condition.

Interestingly, acute administration of FGF1^ΔHBS^ not only reduced the BA biosynthesis by downregulating Cyp7A1 expression, but also suppressed the uptake of BA from hepatic portal vein by reducing the expression of basolateral uptake transporters (Ntcp, Oatp1, and Oatp2) (**Figure 3**). These dual effects can limit the accumulation of BA in the liver and facilitate the protective effects of FGF1 on ANIT-induced cholestasis. To further determine whether the changes of these genes are due to the direct effect of drug stimulation or the negative feedback upon changes in BA content, we measured the content of total BA in mouse liver after single injection. The results showed that synthesis of BA genes was inhibited whereas the hepatic BA content did not change, suggesting that changed expressions of these enzyme and transporters may be directly associated with drug stimulation but not negative feedback.

Although the results presented in the study demonstrate that FGF1^ΔHBS^ suppresses hepatic BA accumulation and protect liver against ANIT-induced injury, several limitations need to be addressed. Firstly, the promiscuity of FGF1 with all FGFRs and its mitogenic activity representing safety concerns hurdles the development of FGF1-based therapy. Although no significant proliferation activity and tumorgenicity in the liver were observed after chronic treatment of FGF1^ΔHBS^, further investigations are needed to comprehensively assess of safety of long-term administration of FGF1^ΔHBS^ or engineer FGF1 variants specifically binding with FGFR4. Secondly, the ANIT-induced intrahepatic cholestaisis mouse model used in the present study only depicts certain aspects of cholestatic liver disease, extra experimental models are also required to evaluate the potentially therapeutic value of FGF1 mutants.

## Conclusion

In conclusion, our study uncovers the association of paracrine FGF1 with BA metabolism and demonstrated that FGF1 functions as a negative regulator of BA synthesis through FGFR4-mediated inhibition of the classical and alternative pathway. Thus, FGF1 can function as a protector against ANIT-induced liver injury under cholestasis. Moreover, the study from engineered FGF1^ΔHBS^ holds a promise of FGF1 to be a therapeutic candidate for cholestatic liver disease or other BA metabolism syndromes.

## Data Availability Statement

All datasets generated for this study are included in the article/**Supplementary Material**.

## Ethics Statement

The animal study was reviewed and approved by the Animal Care and Use Committee of Wenzhou Medical University.

## Author Contributions

HL, CZ, YH, QL, GQ, YW, ZH, and JN researched the data. ZH and JN contributed to the initial discussion and design of the project. HL and JN wrote the manuscript. JN is the guarantor of this work and, as such, had full access to all the data in the study and takes responsibility for the integrity of the data and the accuracy of the data analysis.

## Funding

This work was supported by grants from the Natural Science Foundation of China (81803415, to JN) and the Natural Science Foundation of Zhejiang (LY18H070002 and LQ19H300002, to YW and JW), and the Science and Technology Project of Wenzhou (Y20180176, to JW).

## Conflict of Interest

The authors declare that the research was conducted in the absence of any commercial or financial relationships that could be construed as a potential conflict of interest.
