# Supplementary material for: Paracrine Fibroblast Growth Factor 1 Functions as Potent Therapeutic Agent for Intrahepatic Cholestasis by Downregulating Synthesis of Bile Acid
Source: Front Pharmacol. 2019 Dec 20;10:1515. doi: 10.3389/fphar.2019.01515 (PMC6933012; doi:10.3389/fphar.2019.01515)
Supplement: Supplementary file 1 [file DataSheet_1.pdf]

Supplementary Information

Supplementary Table S1. Primers of genes used for RT-PCR

Gene Name	Forward (5'-3')	Reverse (5'-3')
PCNA	TTTGAGGCACGCCTGATCC	GGAGACGTGAGACGAGTCCAT
Ki67	ATCATTGACCGCTCCTTTAGGT	GCTCGCCTTGATGGTTCCT
AFP	CTTCCCTCATCCTCCTGCTAC	ACAAACTGGGTAAAGGTGATGG
Cyp7A1	GGGATTGCTGTGGTAGTGAGC	GGTATGGAATCAACCCGTTGTC
Cyp27A1	CCAGGCACAGGAGAGTACG	GGGCAAGTGCAGCACATAG
Cyp8B1	CTAGGGCCTAAAGGTTCGAGT	GTAGCCGAATAAGCTCAGGAAG
Cyp7B1	GGAGCCACGACCCTAGATG	GCCATGCCAAGATAAGGAAGC
BSEP	TCTGACTCAGTGATTCTTCGCA	CCCATAAACATCAGCCAGTTGT
MRP2	GTGTGGATTCCCTTGGGCTTT	CACAACGAACACCTGCTTGG
MDR2	CAGCGAGAAACGGAACAGCA	TCAGAGTATCGGAACAGTGTCA
NTCP	CAAACCTCAGAAGGACCAAACA	GTAGGAGGATTATTCCCGTTGTG
OATP1	GTGCATACCTAGCCAAATCACT	CCAGGCCCATAACCACACATC
OATP2	GGGAACATGCTTCGTGGGATA	GGAGTTATGCGGACACTTCTC
β-actin	GTGACGTTGACATCCGTAAAGA	GCCGGACTCATCGTACTCC
FGF1	GGGGAGATCACAACCTTCGC	GTCCCTTGTCCCATCCACG
FGF2	GCGACCCACACGTCAAACTA	TCCCTTGATAGACACAACTCCTC
FGF3	TGCGCTACCAAGTACCACC	CACCGCAGTAATCTCCAGGAT
FGF4	TACCCCGGTATGTTCATGGC	TTACCTTCATGGTAGGCGACA
FGF5	GTACGTGGCCCTGAACAAGA	CGGTGAAGGAAAGTTCCGGT
FGF6	CAGGCTCTCGTCTTCTTAGGC	AATAGCCGCTTTCCCAATTCA
FGF7	TGGGCACTATATCTCTAGCTTGC	GGGTGCGACAGAACAGTCT
FGF8	GGAACCCAGCTGACACTCTC	TCTTCTGCCATGGCGTTGAT
FGF9	ATGGCTCCCTTAGGTGAAGTT	TCCGCCTGAGAATCCCCTTT
FGF10	TTTGGTGTCTTCGTTCCCTGT	TAGCTCCGCACATGCCTTC
FGF16	GTGTTTTCCGGGAACAGTTTGA	GGTGAGCCGTCTTTATTCAGG

FGF17	GCGGCAAATCCGTGAATACC	GGCCGTGTAGTTGTTCTCCA
FGF18	GCCCTGATGTCTGCCAAGTA	CCCTTGGGGTAACGCTTCAT
FGF20	AGGATCACAGTCTCTTCGGTATC	GTCATTCATCCCAAGGTACAGG
FGF22	GGAGATCCGTTCTGTCCGTG	TCCCGGAACCGACCCAT

Supplementary Table S2. Relative mRNA levels of all paracrine FGFs between ANIT-induced cholestasis model and the vehicle group

Gene Name	Vehicle group	ANIT-induced model	P value
FGF1	1.022 ± 0.0551	0.4078 ± 0.0602	<0.0001
FGF2	1.084 ± 0.1451	0.7949 ± 0.0693	0.0938
FGF3	Undetectable	Undetectable	Undetectable
FGF4	1.066 ± 0.129	0.9022 ± 0.2246	0.5385
FGF5	Undetectable	Undetectable	Undetectable
FGF6	1.093 ± 0.1759	1.702 ± 0.6546	0.3845
FGF7	1.201 ± 0.2306	0.9238 ± 0.3158	0.4893
FGF8	Undetectable	Undetectable	Undetectable
FGF9	1.106 ± 0.1361	0.741 ± 0.2351	0.2002
FGF10	1.113 ± 0.1915	0.7422 ± 0.3422	0.3609
FGF16	1.148 ± 0.2165	0.8751 ± 0.3386	0.5081
FGF17	Undetectable	Undetectable	Undetectable
FGF18	1.098 ± 0.1626	1.157 ± 0.2752	0.8580
FGF20	Undetectable	Undetectable	Undetectable
FGF22	Undetectable	Undetectable	Undetectable
